# Skin Barrier Function and Its Importance at the Start of the Atopic March

**DOI:** 10.1155/2012/901940

**Published:** 2012-05-07

**Authors:** Mary Beth Hogan, Kathy Peele, Nevin W. Wilson

**Affiliations:** Section of Allergy, Immunology and Pulmonology, Department of Pediatrics, University of Nevada School of Medicine, Reno, NV 89503, USA

## Abstract

Atopic dermatitis can be due to a variety of causes from nonatopic triggers to food allergy. Control of egress of water and protection from ingress of irritants and allergens are key components of cutaneous barrier function. Current research suggests that a degraded barrier function of the skin allows the immune system inappropriate access to environmental allergens. Epidermal aeroallergen exposure may allow sensitization to allergen possibly initiating the atopic march. Further research into connections between epidermal barrier function and possible allergen sensitization will be important to undertake. Future clinical trials focused on skin barrier protection may be of value as a possible intervention in prevention of the initiation of the atopic march.

## 1. Introduction

The atopic march refers to the natural progression of atopic diseases from atopic dermatitis in infancy to atopic asthma in school age children. Recent research has uncovered exciting data concerning the initiation of the atopic march. A previously little valued component of the epidermis, the strateum corneum, has become an area of scientific attention in the study of the allergic diathesis. This focus on epidermal barrier function potentially provides a heightened understanding of both atopic dermatitis and the initiation of the atopic march. Improving barrier function with reduced water loss and minimized ingress of allergens might become an important tool to controlling the onset of the atopic march.

## 2. Epidemiology and Definition of Atopic Dermatitis

Atopic dermatitis is one of the most significant and common skin diseases of childhood. Studies from Japan suggest that the prevalence for atopic dermatitis in childhood may be as high as 11–25% [1, 2]. There is a 15.8% prevalence of atopic dermatitis in 3–5-year-old children in New Zealand [3]. A US-population-based study revealed that the prevalence of atopic dermatitis amongst 5–9-year olds was 17.2% [4, 5].

Atopic dermatitis is an inflammatory cutaneous disease characterized by erythema, pruritus, altered barrier function, and immune dysfunction resulting in IgE sensitization. Dysfunction of antimicrobial peptides such as defensins, psoriasins, and cathelicidins is associated with the development of atopic dermatitis [6–8]. Functional alteration of these peptides has been associated with eczema herpeticum. A perturbation in the function of these peptides can result in cutaneous infection with *Staphylococcus aureus*. Infectious sequelae frequently result in atopic dermatitis exacerbations. These and other developments in atopic dermatitis are exciting. However, the emphasis of this paper will be confined to defining the importance of altered strateum corneum function and its possible link to the atopic march.

## 3. Components of the Strateum Corneum Establishing Skin Barrier Function

**Figure 1:**
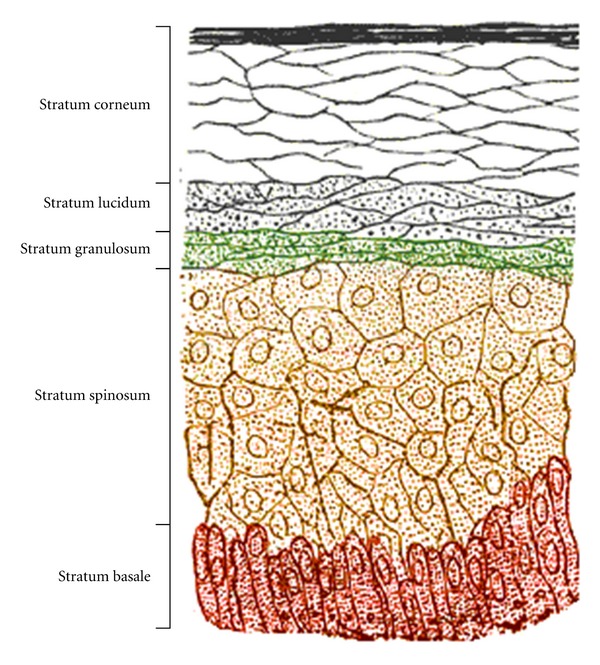
Layers forming the protective epidermal barrier.

Epidermal layers of the skin include, the stratum corneum, stratum lucidum, stratum granulosum, stratum spinosum, and stratum basale. The structure of these layers is demonstrated in Figure 1 [9]. Until recently, the outermost layer of the dermis was relatively ignored as a factor in the development of atopic dermatitis. In the epidermis there are multiple components important to barrier function. These components include claudin, desmoglein, filaggrin, ceramide, and proper control of proteases (Table 1). When properly functioning, this layer prevents water loss and provides a barrier to epidermal invasion of allergens and bacteria.

Each corneocyte in the stratum corneum is held together by tight junctions and scaffolding proteins. Claudins are a family of proteins that are important components of the tight junctions between corneocytes that help to maintain the skin barrier. Claudin-deficient patients have aberrant formation of tight junctions that cause disruption of the bioelectric barrier [10]. Claudins are an essential component controlling the paracellular barrier flow of molecules in the intercellular space between the cells of an epithelium. These tight junctions help prevent moisture loss through this layer of the skin as well as block access through the skin of external environmental allergens. Claudin expression in atopic dermatitis patients has been inversely correlated to increased T_H_2 biomarker expression [10]. This suggests that claudin may help inhibit immune exposure to allergic stimuli.

Similar tight junction dysfunction has been found in desmoglein transgenic mice. Desmogleins play a role in the formation of desmosomes that promotes cell-to-cell tight junction adhesion in the stratum corneum. Mice without desmoglein were ultimately found to die from dehydration presumably due to increased transepidermal water loss mediated by lack of corneocyte adhesion [11].

A different genetic knock-out mouse model of atopic dermatitis examined the relationship between scaffolding proteins and skin barrier function. Scaffolding proteins are required to overlaying tight junction linked corneocytes with cross-linked proteins and lipids to form an effective epidermal barrier. Loss of epidermal scaffolding proteins such as involucrin, envoplakin, and periplakin is associated with alterations in epidermal barrier function such as filaggrin and desmoglein-1 processing with formation of an abnormal cornified epidermal envelope [12]. Immune regulatory dysfunction after disruption of scaffolding proteins was associated with increased CD4+ T cell infiltration and lack of gamma delta+ T cells. This association suggests that an abnormal epidermal layer may contribute to the allergic inflammatory process associated with atopic dermatitis.

Another factor allowing proper function of the epidermis is the control of the on or off activity of skin proteases. SPINK is a protein that inhibits serine protease action in the skin. The SPINK gene is absent in Netherton's syndrome. This syndrome is characterized by severe atopic dermatitis, scaling, and an elevated serum IgE [13]. In this potentially lethal disease, lack of SPINK results in uncontrolled serine protease elastase-2 activity. Increased protease activity negatively alters filaggrin and lipid (ceramide) processing thereby decreasing skin barrier function. It has been suggested that barrier function in populations with SNP alterations of SPINK5 may lead to increased susceptibility to asthma [14].

Increased protease functioning also occurs in atopic dermatitis patients. Allergens such as dust mite, cockroach and mold can activate serine proteases, adversely affecting the epidermal barrier [15]. In fact, dust mite and cockroach allergens themselves can be proteolytically active and stimulate the serine protease pathway thereby decreasing skin barrier function [16, 17].

Filaggrin is an important protein found in lamellar bodies of stratum granulosum corneocytes. When these granules are released they become a vital component of the extracellular matrix of the stratum corneum. Mutations of the filaggrin gene have been associated with ichthyosis vulgaris and persistent atopic dermatitis [18, 19]. Filaggrin gene defects may exist in as many as 50% of atopic dermatitis patients [20, 21]. Meta-analysis of filaggrin polymorphism data has identified a genetic alteration in filaggrin as a significant risk for development of atopic dermatitis [22]. The results of filaggrin gene mutations are striking as several studies have demonstrated that the severity of atopic dermatitis correlates with the number of filaggrin gene defects [23–26].

## 4. Permeability Layer Disruption Is Bidirectional Allowing Both Epidermal Water Loss and Allergen Penetration

The lipid lamellar matrix is an integral component in controlling the barrier function of the skin. Decreased protein-bound omega-hydroxyceramides are found in the lesional skin of atopic dermatitis patients as compared to control patients [27]. In this study, deficiency of barrier lipid free ceramides was determined to be a major contributing factor in damaging the permeability barrier of the skin.

A murine model of atopic dermatitis illustrates the importance of ceramide in preventing allergen-induced atopic dermatitis. In this model, a synthetic ceramide was applied to the skin of mice noted for the ability to develop atopic dermatitis [28]. In this model of dust-mite-induced atopic dermatitis, ceramide application reduced skin thickness and actually blocked components of allergic inflammation. Inflammatory factors prevented were the cutaneous infiltration by mast cells and expression of tumor necrosis factor. An *in vitro* study has demonstrated that ceramide inhibits mast cell production of IL-5, IL-10, and IL-13 [29]. These models suggest that an intact lipid matrix in the strateum corneum may actually prevent epidermal penetration of allergen and allergic atopic dermatitis.

Filaggrin is another key protein in protecting against water loss through the strateum corneum and is present in the epidermis as early as 3 months of age [30]. Filaggrin-deficient atopic dermatitis patients have decreased filaggrin-derived natural moisturization factors [31, 32]. The function of filaggrin in the development of the epidermal barrier has been confirmed in murine models of atopic dermatitis. Scharschmidt et al. describe altered lamellar body secretion in an atopic dermatitis flaky tailed mouse model. In this model, a decrease in stratum corneum extracellular matrix component correlated with increased permeability of water soluble molecules [33].

Degradation products of filaggrin have been found to contribute to the formation of the epidermal barrier by providing acidity. This critical function of acidification and hydration of the skin has been linked to filaggrin gene defects in atopic dermatitis [34]. *In vitro* models of skin demonstrate that urocanic acid is a key filaggrin-derived amino acid linked to skin acidity [35]. In this study, skin pH became more basic with decreased filaggrin, which was associated with an increase in dye penetration. The skin performs a vital function in providing barrier function, and if this is interrupted by filaggrin deficiency, then inflammatory irritants, haptens, and infections have greater access to the deeper layers of the skin.

## 5. Barrier Proteins May Be One Line of Defense against Allergic Diseases

In studies of filaggrin-deficient children, increased transepidermal water loss along with development of specific IgE antibody to dust mite and cat was found with asthma [36, 37]. A recent meta-analysis confirmed that the risk of developing asthma was increased in those with eczema but not in those without atopic dermatitis. Filaggrin gene mutations were linked directly to atopic dermatitis, allergic rhinitis, and the development of asthma in children [38, 39]. These studies together suggest that the lack of barrier function itself may contribute to the development of allergy and asthma.

This reduction in barrier function may allow for the development of inflammation due to increased penetration of allergen through the skin allowing IgE sensitization. In a filaggrin-deficient murine model, allergen exposure over lesional skin was linked to the development of allergen-specific IgE and Th17 expression [40]. Facilitation of allergen sensitization in individuals with filaggrin deficiency is believed to be due to reduced barrier function. This hypothesis of increased skin penetrability correlating with diminished barrier function has been tested with both water and lipid soluble dyes *in vivo*. Dye penetration was deepest with severity of atopic dermatitis and correlated to increasing serum IgE as compared to control patients [34, 35].

## 6. The Atopic March

Children with atopic dermatitis have a significant risk of going on to develop other atopic diseases such as allergic sensitization and asthma. Whether eczema precedes or postdates the development of allergic sensitization is not clear for all children [41]. Thirty to fifty percent of the children who develop atopic dermatitis go on to develop asthma and two-thirds go on to have allergic rhinitis [36, 42]. In one study of 169 infants with eczema, 35% developed subspecialist-diagnosed asthma and these children had inhalant-induced allergy [36].

The allergic march has a pattern of allergic sensitization that changes as children age. In a study with 262 children with atopic dermatitis, IgE sensitization to food was common under 2 years of age. Between 2 and 5 years of age the children had food allergy but also started to develop inhalant allergy. After 5 years of age the children had mostly inhalant allergy as a significant allergic factor associated with their atopic dermatitis [43].

Dust mite and cockroach are allergens associated with atopic dermatitis [44]. *Alternaria* allergy eventually develops in 56% of atopic dermatitis children by 12 years of age [45]. When 30 children with atopic dermatitis were compared with 30 patients with respiratory symptoms without atopic dermatitis, aeroallergen allergy was significant in a selected population of atopic dermatitis patients. Of these children with atopic dermatitis, 70% were skin test positive for mite, 70% were positive for cockroach, 63% were skin test positive to house dust, 50% had evidence of IgE sensitization to mold, and 43% had IgE reactivity to grass. However, only 10 percent of the kids with respiratory symptoms (and no atopic dermatitis) were allergic to any aeroallergen in this study [46]. This suggests an important link between atopic dermatitis and development of inhalant allergy rather than inhalation of allergen resulting in allergic sensitization and asthma.

Studies suggest that a significant number of these children develop atopic dermatitis first and subsequently become sensitized to aeroallergens. Allergic rhinitis and asthma then follow. Suggested risk factors for this chain of events include atopic parents, possibly cat ownership [47], and presence of eczema prior to 4 years of age [48]. The progression to asthma is as high as 29.5% in children with eczema [49]. Cumulative smoke exposure may be an additional risk factor influencing the development of asthma in FLG-null patients [50]. A recent genome-wide study found that FLG mutation was associated with a chromosomal (11q13.5) variant. These individuals had increased risk of developing atopic dermatitis associated with asthma [51]. This risk of atopic dermatitis patients then developing asthma is significant enough that the Asthma Predictive Index utilizes the presence of atopic dermatitis in a wheezing infant as one of two major criteria for predicting eventual asthma [52].

## 7. Therapeutic Options Involving Barrier Function and Atopic Dermatitis

Identification of ceramide as a critical element in barrier function has led to the development of ceramide-based emollients. In a study of mild-moderate atopic dermatitis patients, application of ceramide emollient resulted in 69% of patients having no symptoms of eczema and skin hydrations scores improving significantly [53]. Ceramide-dominant lipid-based emollient was used in 24 children with stubborn atopic dermatitis. In this study, skin cohesion and transepidermal water loss levels improved with atopic dermatitis scores as a result of the reestablishment of extracellular lamellar membranes in the stratum corneum [54]. As in murine studies, ceramide-based emollients in humans have been shown to decrease inflammatory cytokine interleukin-4 expression and decrease transepidermal water loss in atopic dermatitis patients [55].

Strategies for possible prevention of atopic dermatitis include encouraging the natural acidification of the skin. Treating mice with lactobionic acid was associated with normal barrier function in addition to normalization of atopic inflammatory markers such as serum IgE [56]. These atopic dermatitis mice achieved normal lamellar body secretion and lipid bilayer formation after lactobionic acid treatment only. In human patients, early studies suggest that neutral pH soaps may be an effective therapeutic component when treating atopic dermatitis [57]. Whether neutral “baby washes” or water washing alone after birth protects the epidermal barrier and prevents the onset of atopic dermatitis remains to be studied.

Immediate barrier repair can even be implemented on a delipidized stratum corneum with petrolatum without application of ceramide [58]. A preliminary study investigated whether petrolatum can be used immediately after birth to prevent atopic dermatitis. High-risk neonates with first-degree relatives having atopic dermatitis or asthma were enrolled at birth in a feasibility of prevention study [59]. The study utilized an emollient cream and rescue petrolatum at birth as a prevention of atopic dermatitis. This population historically has a 30–50% risk of developing atopic dermatitis by 2 years of age. Only 15% of these treated infants developed eczema and barrier function measurements reflective of normal skin, which suggests a protective effect.

## 8. Questions for the Future

Murine models suggest that allergen penetration through the skin barrier is important to the development of atopic dermatitis. Human studies show a link between barrier protein dysfunction and development of atopic dermatitis and possibly asthma. Clinical trials have not yet directly looked at allergen penetration through a disordered skin barrier and subsequent development of asthma. Controlled trials with a significant number of patients would be important in determining whether prevention strategies for decreasing allergic sensitization and atopic dermatitis are effective. Longer-term follow-up studies determining whether the onset of allergic rhinitis and asthma is decreased by decreasing the incidence of eczema would also be exciting.

It is apparent that not all patients with filaggrin deficiency go on to develop atopic dermatitis [60]. In fact, if they do develop eczema, remission is possible. It has not been studied whether these patients also are the same atopic dermatitis patients who do not have disease progression to asthma. Factors determining nonexpression of allergic rhinitis and asthma have not been elucidated. Which populations are destined to have a milder disease course? Patients may “naturally" modify disease expression such as self-treatment with hydration and if they do can it be applied as prevention in high-risk infants? Which genetic modifiers associated with filaggrin deficiency lead to or prevent the atopic march?

Research in the alteration and prevention of loss of stratum corneum barrier function may provide answers to questions raised regarding the development of atopic dermatitis and asthma.  Early identification of at-risk individuals and developing treatment strategies allowing retention of good skin barrier function may assist in the prevention of allergic sensitization in some individuals. The atopic march may not be inevitable for certain genetically predisposed individuals. Research into maintenance of barrier function in patients at risk for atopy may elucidate the ability to control skin barrier function and prevent onset of atopic dermatitis, and, hopefully, asthma.

## Figures and Tables

**Table 1:** Functions of epidermal barrier components and possible protective role in prevention of atopic march.

Barrier component	Type	Function	Possible role in preventing atopic march
Claudin	Tight junction protein	Prevention of water loss	Prevention of T_H_2 activation
Desmoglein	Desmosome formation	Prevention of water loss	Blocking allergen penetration
Involucrin/envoplakin/periplakin	Scaffolding protein	Structural components to create epidermal barrier	Allowing appropriate immunoregulatory T-cell environment
Urocanic acid	Chromophore	Hygroscopic acid-base regulator/photoprotection	Maintaining skin barrier function
Filaggrin	Protein	Decreased permeability of water soluble molecules/epidermal differentiation	Blocking allergen penetration
Ceramide	Lipid	Contribution to skin permeability barrier and epidermal differentiation	Blocking mast cell infiltration/expression of TNF Blocking Mast production of allergic cytokines. Blocking allergen penetration
Skin protease inhibitors (SPINK)	Protein	Prevention of protease alteration in filaggrin and ceramide production	Maintaining skin barrier function

## References

[1] Kay J, Gawkrodger DJ, Mortimer MJ, Jaron AG (1994). The prevalence of childhood atopic eczema in a general population. *Journal of the American Academy of Dermatology*.

[2] Shamssain MH, Shamsian N (1999). Prevalence and severity of asthma, rhinitis, and atopic eczema: the north east study. *Archives of Disease in Childhood*.

[3] Purvis DJ, Thompson JMD, Clark PM (2005). Risk factors for atopic dermatitis in New Zealand children at 3.5 year of age. *British Journal of Dermatology*.

[4] Laughter D, Istvan JA, Tofte SJ, Hanifin JM (2000). The prevalence of atopic dermatitis in Oregon schoolchildren. *Journal of the American Academy of Dermatology*.

[5] Spergel JM (2010). Epidemiology of atopic dermatitis and atopic March in children. *Immunology and Allergy Clinics of North America*.

[6] Hata TR, Kotol P, Boguniewicz M (2010). History of eczema herpeticum is associated with the inability to induce human *β*-defensin (HBD)-2, HBD-3 and cathelicidin in the skin of patients with atopic dermatitis. *British Journal of Dermatology*.

[7] Gläser R, Meyer-Hoffert U, Harder J (2009). The antimicrobial protein psoriasin (S100A7) is upregulated in atopic dermatitis and after experimental skin barrier disruption. *Journal of Investigative Dermatology*.

[8] Ong PY, Ohtake T, Brandt C, Strickland I (2002). Endogenous antimicrobial peptides and skin infections in atopic dermatitis. *The New England Journal of Medicine*.

[9] Marks JG, Miller JJ (2006). *Lookingbill and Marks' Principles of Dermatology*.

[10] De Benedetto A, Rafaels NM, McGirt LY (2011). Tight junction defects in patients with atopic dermatitis. *Journal of Allergy and Clinical Immunology*.

[11] Elias PM, Matsuyoshi N, Wu H (2001). Desmoglein isoform distribution affects stratum corneum structure and function. *Journal of Cell Biology*.

[12] Sevilla LM, Nachat R, Groot KR (2007). Mice deficient in involucrin, envoplakin, and periplakin have a defective epidermal barrier. *Journal of Cell Biology*.

[13] Bonnart C, Deraison C, Lacroix M (2010). Elastase 2 is expressed in human and mouse epidermis and impairs skin barrier function in Netherton syndrome through filaggrin and lipid misprocessing. *Journal of Clinical Investigation*.

[14] Liu Q, Xia Y, Zhang W (2009). A functional polymorphism in the SPINK5 gene is associated with asthma in a Chinese Han Population. *BMC Medical Genetics*.

[15] Jeong SK, Kim HJ, Youm JK (2008). Mite and cockroach allergens activate protease-activated receptor 2 and delay epidermal permeability barrier recovery. *Journal of Investigative Dermatology*.

[16] Roelandt T, Heughebaert C, Hachem JP (2008). Proteolytically active allergens cause barrier breakdown. *Journal of Investigative Dermatology*.

[17] Mascia F, Mariani V, Giannetti A, Girolomoni G, Pastore S (2002). House dust mite allergen exerts no direct proinflammatory effects on human keratinocytes. *Journal of Allergy and Clinical Immunology*.

[18] Sandilands A, O’Regan GM, Liao H (2006). Prevalent and rare mutations in the gene encoding filaggrin cause ichthyosis vulgaris and predispose individuals to atopic dermatitis. *Journal of Investigative Dermatology*.

[19] Barker JN, Palmer CN, Zhao Y (2007). Null mutations in the filaggrin gene (FLG) determine major susceptibility to early-onset atopic dermatitis that persists into adulthood. *Journal of Investigative Dermatology*.

[20] O’Regan GM, Irvine AD (2010). The role of filaggrin in the atopic diathesis. *Clinical and Experimental Allergy*.

[21] Jungersted JM, Scheer H, Mempel M (2010). Stratum corneum lipids, skin barrier function and filaggrin mutations in patients with atopic eczema. *Allergy*.

[22] Baurecht H, Irvine AD, Novak N (2007). Toward a major risk factor for atopic eczema: meta-analysis of filaggrin polymorphism data. *Journal of Allergy and Clinical Immunology*.

[23] Palmer CN, Irvine AD, Terron-Kwiatkowski A (2006). Common loss-of-function variants of the epidermal barrier protein filaggrin are a major predisposing factor for atopic dermatitis. *Nature Genetics*.

[24] Nomura T, Sandilands A, Akiyama M (2007). Unique mutations in the filaggrin gene in Japanese patients with ichthyosis vulgaris and atopic dermatitis. *Journal of Allergy and Clinical Immunology*.

[25] Ruether A, Stoll M, Schwarz TT, Schreiber S, Folster-Holst R (2006). Filaggrin loss-of-function variant contributes to atopic dermatitis risk in the population of Northern Germany. *British Journal of Dermatology*.

[26] Brown SJ, Relton CL, Liao H (2009). Filaggrin haploinsufficiency is highly penetrant and is associated with increased severity of eczema: further delineation of the skin phenotype in a prospective epidemiological study of 792 school children. *British Journal of Dermatology*.

[27] Macheleidt O, Kaiser HW, Sandhoff K (2002). Deficiency of epidermal protein-bound *ω*-hydroxyceramides in atopic dermatitis. *Journal of Investigative Dermatology*.

[28] Kang JS, Yoon WK, Youm JK (2008). Inhibition of atopic dermatitis-like skin lesions by topical application of a novel ceramide derivative, K6PC-9p, in NC/Nga mice. *Experimental Dermatology*.

[29] Chiba N, Masuda A, Yoshikai Y, Matsuguchi T (2007). Ceramide inhibits LPS-induced production of IL-5, IL-10, and IL-13 from mast cells. *Journal of Cellular Physiology*.

[30] Flohr C, England K, Radulovic S (2010). Filaggrin loss-of-function mutations are associated with early-onset eczema, eczema severity and transepidermal water loss at 3 months of age. *British Journal of Dermatology*.

[31] Kezic S, O'Regan GM, Yau N (2011). Levels of filaggrin degradation products are influenced by both filaggrin genotype and atopic dermatitis severity. *Allergy*.

[32] Nemoto-Hasebe I, Akiyama M, Nomura T, Sandilands A, McLean WH, Shimizu H (2009). Clinical severity correlates with impaired barrier in filaggrin-related eczema. *Journal of Investigative Dermatology*.

[33] Scharschmidt TC, Man MQ, Hatano Y (2009). Filaggrin deficiency confers a paracellular barrier abnormality that reduces inflammatory thresholds to irritants and haptens. *Journal of Allergy and Clinical Immunology*.

[34] Knor T, Meholji-Fetahovic A, Mehmedagic A (2011). Stratum corneum hydration and skin surface pH in patients with Atopic dermatitis. *Acta Dermatovenerologica Croatica*.

[35] Mildner M, Jin J, Eckhart L (2010). Knockdown of filaggrin impairs diffusion barrier function and increases UV sensitivity in a human skin model. *Journal of Investigative Dermatology*.

[36] Spergel JM, Paller AS (2003). Atopic dermatitis and the atopic march. *Journal of Allergy and Clinical Immunology*.

[37] Henderson J, Northstone K, Lee SP (2008). The burden of disease associated with filaggrin mutations: a population-based, longitudinal birth cohort study. *Journal of Allergy and Clinical Immunology*.

[38] Ohshima Y, Yamada A, Hiraoka M (2002). Early sensitization to house dust mite is a major risk factor for subsequent development of bronchial asthma in Japanese infants with atopic dermatitis: results of a 4-year follow-up study. *Annals of Allergy, Asthma and Immunology*.

[39] van den Oord R, Sheikh A (2009). Filaggrin gene defects and risk of developing allergic sensitisation and allergic disorders: systematic review and meta-analysis. *BMJ*.

[40] Oyoshi MK, Murphy GF, Geha RS (2009). Filaggrin-deficient mice exhibit TH17-dominated skin inflammation and permissiveness to epicutaneous sensitization with protein antigen. *Journal of Allergy and Clinical Immunology*.

[41] Osawa R, Akiyama M, Shimizu H (2011). Filaggrin gene defects and the risk of developing allergic disorders. *Allergology International*.

[42] Spergel JM (2010). Epidemiology of atopic dermatitis and atopic March in children. *Immunology and Allergy Clinics of North America*.

[43] Wang IJ, Lin YT, Yang YH (2004). Correlation between age and allergens in pediatric atopic dermatitis. *Annals of Allergy, Asthma and Immunology*.

[44] Michel S, Yawalkar N, Schnyder B, Fischer B, Helbling A (2009). Eczematous skin reaction to atopy patch testing with cockroach in patients with atopic dermatitis. *Journal of Investigational Allergology and Clinical Immunology*.

[45] Hedayati MT, Arabzadehmoghadam A, Hajheydari Z (2009). Specific IgE against Alternaria alternata in atopic dermatitis and asthma patients. *European Review for Medical and Pharmacological Sciences*.

[46] Wananukul S, Huiprasert P, Pongprasit P (1993). Eczematous skin reaction from patch testing with aeroallergens in atopic children with and without atopic dermatitis. *Pediatric Dermatology*.

[47] Lowe AJ, Abramson MJ, Hosking CS (2007). The temporal sequence of allergic sensitization and onset of infantile eczema. *Clinical and Experimental Allergy*.

[48] Epstein TG, Bernstein DI, Levin L (2010). Opposing effects of cat and dog ownership and allergic sensitization on eczema in an atopic birth cohort. *Journal of Pediatrics*.

[49] van der Hulst AE, Klip H, Brand PL (2007). Risk of developing asthma in young children with atopic eczema: a systematic review. *Journal of Allergy and Clinical Immunology*.

[50] Berg ND, Husemoen LL, Thuesen BH (2012). Interaction between filaggrin null mutations and tobacco smoking in relation to asthma. *Journal of Allergy and Clinical Immunology*.

[51] Marenholz I, Bauerfeind A, Esparza-Gordillo J (2011). The eczema risk variant on chromosome 11q13 (rs7927894) in the population-based ALSPAC cohort: a novel susceptibility factor for asthma and hay fever. *Human Molecular Genetics*.

[52] Castro-Rodriguez JA, Holberg CJ, Wright AL, Martinez FD (2000). A clinical index to define risk of asthma in young children with recurrent wheezing. *American Journal of Respiratory and Critical Care Medicine*.

[53] Na JI, Hwang JS, Park HJ (2010). A new moisturizer containing physiologic lipid granules alleviates atopic dermatitis. *Journal of Dermatological Treatment*.

[54] Chamlin SL, Kao J, Frieden IJ (2002). Ceramide-dominant barrier repair lipids alleviate childhood atopic dermatitis: changes in barrier function provide a sensitive indicator of disease activity. *Journal of the American Academy of Dermatology*.

[55] Park KY, Kim DH, Jeong MS, Li K, Seo SJ (2010). Changes of antimicrobial peptides and transepidermal water loss after topical application of tacrolimus and ceramide-dominant emollient in patients with atopic dermatitis. *Journal of Korean Medical Science*.

[56] Bikowski J (2001). The use of cleansers as therapeutic concomitants in various dermatologic disorders. *Cutis*.

[57] Simpson EL, Berry TM, Brown PA, Hanifin JM (2010). A pilot study of emollient therapy for the primary prevention of atopic dermatitis. *Journal of the American Academy of Dermatology*.

[58] Lodén M (2003). Role of topical emollients and moisturizers in the treatment of dry skin barrier disorders. *American Journal of Clinical Dermatology*.

[59] Hatano Y, Man MQ, Uchida Y (2009). Maintenance of an acidic stratum corneum prevents emergence of murine atopic dermatitis. *Journal of Investigative Dermatology*.

[60] Thyssen J, Carlsen B, Bisgaard H (2012). Individuals who are homozygous for the 2282del4 and R501X filaggrin null mutations do not always develop dermatitis and complete long-term remission is possible. *Journal of the European Academy of Dermatology and Venereology*.

